# A Vacuum Waveguide Filter Bank Spectrometer for Far-Infrared Astrophysics

**DOI:** 10.1007/s10909-024-03166-2

**Published:** 2024-06-05

**Authors:** Rong Nie, Jeffrey Filippini, Elyssa Brooks, Peter Barry, Jake Connors, Marcin Gradziel, Dale Mercado, Vesal Razavimaleki, Erik Shirokoff, Locke Spencer, Serena Tramm, Neil Trappe, Michael Zemcov

**Affiliations:** 1 Department of Physics, University of Illinois Urbana-Champaign, Urbana, IL 61821 USA; 2 Department of Astronomy and Astrophysics, University of Chicago, Chicago, IL 60637 USA; 3 School of Physics and Astronomy, Cardiff University, Cardiff, CF24 3AA UK; 4 National Institute of Standards and Technology, Boulder, CO 80305 USA; 5 Experimental Physics Department, Maynooth University, Maynooth, Ireland; 6 School of Physics and Astronomy, Rochester Institute of Technology, Street, City, NY 14623 USA; 7 Department of Physics and Astronomy, University of Lethbridge, Lethbridge, AB T1K 3M4 Canada

**Keywords:** Far-infrared, MKID, Terahertz, Spectroscopy

## Abstract

Traditional technologies for far-infrared (FIR) spectroscopy generally involve bulky dispersive optics. Integrated filter bank spectrometers promise more compact designs, but implementations using superconducting transmission line networks become lossy at terahertz frequencies. We describe a novel on-chip spectrometer architecture designed to extend this range. A filter bank spectrometer is implemented using vacuum waveguide etched into a silicon wafer stack. A single trunk line feeds an array of resonant cavities, each coupled to a kinetic inductance detector fabricated on an adjacent wafer. We discuss the design and fabrication of a prototype implementation, initial test results at ambient temperature, and prospects for future development.

## Introduction

Spectroscopic observations at far-infrared (FIR) wavelengths (${\sim }$30–1000 $\mu$m) can offer invaluable insights into the cold and dusty universe, thanks to a rich array of diagnostic emission and absorption features from atomic and molecular species. Future FIR missions (e.g., [1]) will demand compact, low-noise spectrometer solutions that place arrays of thousands of sensors onto the sky from above Earth’s atmosphere. A variety of well-understood FIR spectrometer architectures are available for achieving resolving powers $R = \lambda /\Delta \lambda$ in the $10^{2} {-} 10^{4}$ range; examples from recent space missions include grating spectrometers (*Herschel*-PACS [2]), Fourier transform spectrometers (FTS) (*Herschel* SPIRE-FTS [3]), and Fabry-Perot etalons (ISO SWS/LWS [4, 5]). All of these require relatively bulky dispersive optics that are challenging to scale to high pixel counts and large fields of view within the constraints of a space mission.

Integrated filter banks are a promising architecture for compact imaging spectrometers. In this approach, power from a single transmission line is coupled to a series of tuned resonators, each delivering a specific narrow bandwidth to a dedicated low-noise detector. Filter banks with $R \gtrsim 100$ have been implemented at 200-440 GHz using networks of superconducting microstrip or coplanar waveguide, notably by the SuperSpec [6–8]) and DESHIMA [9–11] teams. Extensions of these and other superconducting spectrometer solutions (e.g., [12, 13]) to shorter FIR wavelengths are limited by loss due to quasiparticle production above the superconducting gap energy. Nb-based designs are thus limited to $\lambda \gtrsim 450~\mu$m, though future development using higher-$T_c$ materials (e.g., NbN, NbTiN, MgB_2_) may relax this constraint somewhat.

Hollow waveguide is widely used at microwave frequencies due to its lower loss relative to other transmission lines at fixed surface resistance. In this paper, we describe an implementation of a filter bank spectrometer using a vacuum waveguide network etched into a silicon wafer. Our approach is similar to that of WSPEC [14, 15], which employs machined metal waveguide; here we use modern micromachining techniques to extend this architecture to higher frequencies. Here we describe development and prototyping efforts, and progress toward a fieldable device. Further information on the detector arrays is given elsewhere in these proceedings [16].

## Device Concept

**Fig. 1:**
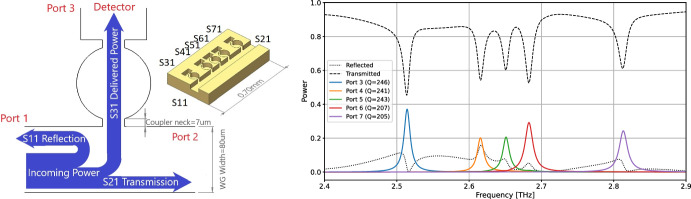
*Left:* Schematic drawing of a single 2.5 THz resonator, showing labeled power flows for a single resonator. Inset shows a 5-port test device with labeled ports. *Right:* HFSS simulation of power flows in this 5-channel device, showing the fraction of incident power delivered to each port. We assume a gold surface with RRR=10, 40 nm r.m.s. roughness, and no wafer gap

Our initial device concept is illustrated in Fig. 1. A rectangular trunk waveguide is coupled to a series of circular cavities, each tuned to a different resonant frequency by changes in shape and coupling. As power from a feed travels down the trunk line, each resonator peels off power in a single narrow band and delivers it to a dedicated low-noise kinetic inductance detector (KID). In order to ensure low loss, we use vacuum waveguide coated with a high-conductivity metal film (e.g. gold) and operated at cryogenic temperatures. The etched assembly is bonded to a metal-coated lid wafer to complete the waveguide boundary. A single micro-cavity resonator of similar architecture has been demonstrated in [17] at room temperature, with low resonant quality factors (*Q*) and no output channel. Our initial development goal is a 5-channel device targeting $R\sim 100$ at $\lambda = 120~\mu$m (2.5 THz), but this design is straightforwardly scalable to a wide range of wavelengths.

Achievable spectrometer performance is largely determined by the intrinsic quality factor, $Q_i$, of the cavity resonator. For ambient temperature gold walls at 2.5 THz, we expect $Q_i\sim 500$ [18]. This should increase with conductivity at cryogenic temperatures, limited by film quality, the anomalous skin effect [19], surface roughness, and any loss at the boundary between lid and waveguide wafers.

**Fig. 2:**
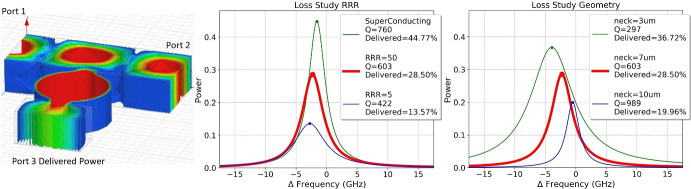
*Left:* Simulated electric field distribution for a resonator and waveguide driven on-resonance. *Center:* Simulated power delivered to the detector port as a function of frequency (expressed relative to the 2.53 THz design resonance) for various values of surface resistivity (ideal lossless, alongside gold of two RRR values); powers are measured relative to that driving the waveguide (caption indicates peak transmission). *Right:* Same, for varying dimensions of the neck that couples resonator to trunk

We optimized our filter bank design using simulations of 1- to 5-resonator structures using Ansys HFSS. In addition to physical dimensions, these simulations vary surface resistivity (parameterized by RRR: the residual resistance ratio relative to room temperature), surface r.m.s., and a possible gap between wafers. Proof-of-principle simulations of a nominal structure (Fig. 1, right) indicate promising performance. By tuning the geometries of the couplings ($Q_c$) between resonator, waveguide, and detector, we can trade overall *Q* (and thus resolving power) against optical efficiency (Fig. 2). Efficiencies of ${\gtrsim }20\%$ for the resolving structure at $R\gtrsim 100$ seem plausible, which should be competitive in combination with the compact architecture.

Power delivered to each resonator is measured using a low-noise, low-volume, lumped-element KID. Each KID consists of an inter-digitated capacitor coupled to a simple loop inductor; the latter doubles as a waveguide probe to couple power to the KID. KIDs and associated readout wiring are patterned on the lid wafer, with care to maintain distance between the KID capacitor and lossy metals. Further information on the detector arrays and packaging are given in [16].

## Process Development

**Fig. 3:**
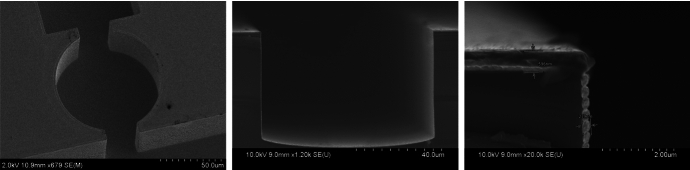
SEM images of a 2.7 THz resonator cavity. *Left:* Cavity top view, with trunk line at bottom; *Center:* Cross section of cavity. *Right:* Corner detail, showing gold thickness (596 nm on horizontal surfaces, 239 nm on vertical walls) and beading

Waveguide structures are defined using deep reactive-ion etching (DRIE) into a silicon wafer. This technique produces precisely defined vertical walls, with shallow “scalloping” associated with individual etch steps. We begin with high-resistivity float-zone wafers, to enable wafer alignment under an infrared microscope. Etched devices (Fig. 3) show surface r.m.s. in the 10 s of nm, ${<}0.1\%$ of the target wavelength. If desired, further smoothing can be achieved using a wet oxide furnace step and HF dip, as described in [20]. The waveguide bottom surface generally shows modest curvature; this will be addressed in later devices by using the oxide layer of a silicon-on-insulator (SOI) wafer to define the depth of the waveguide trench.

After etching, the waveguide walls are coated with sputtered gold at thicknesses much greater than the skin depth (${\sim }80$ nm in Au at 1 THz and 300 K). We find that the deposited gold is thinner along vertical walls relative to horizontal surfaces. We also find significant “beading” of the gold along the side walls, especially if the gold is annealed after deposition. Both can be addressed with thicker gold layers and perhaps by additional smoothing of the etched surface as described above.

We further tested several processes for deposition of the gold layer, making sample wafers with several combinations of sputtering temperature, thickness, adhesion layer, and a possible annealing step. For each sample, we performed 4 Kelvin DC resistance measurements as a proxy for film resistivity. The lowest-loss gold films (RRR$\sim 12$) were achieved with no adhesion layer and a post-sputtering annealing step, but these showed poor mechanical adhesion. A Ti adhesion layer increased loss (RRR<3), especially after annealing, likely due to diffusion into the gold; initial tests with a Pd adhesion layer are more promising (RRR$\sim 4$).

## Filter Bank Test Device

**Fig. 4:**
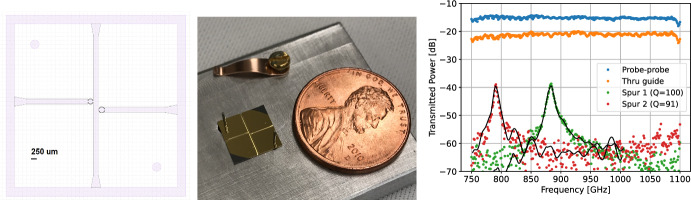
*Left:* Four-port test device mask, with through ports at top and bottom, resonator ports to left and right, and sectoral horns on all ports. *Center:* Photograph of the completed test wafer, without lid. *Right:* Measured test device transmissions from input to all three output ports (straight waveguide and two resonator spurs), and between the bare VNA probes. The normalization of the overlaid whole-device model is adjusted slightly to match data, due to coupling uncertainty

In order to test basic stack assembly and waveguide performance, we constructed and tested a simple 4-port test device (Fig. 4). Two resonators tap off from a single trunk line crossing the wafer, with all ports coupled to free space using simple *H*-plane planar sectoral horns for ease of fabrication. The lid wafer is gold-coated but unpatterned. The resonators are weakly coupled ($Q_c\sim 2000$) to better isolate the resonator performance ($Q \sim Q_i$). The stack is aligned with two dowel pins and clamped together by a spring assembly. This device was scaled to ${\sim }0.9$ THz to enable characterization at room temperature with a vector network analyzer (VNA) at Maynooth University.

Initial test results for the 4-port device are shown in Fig. 4. Both resonators are clearly visible, with peak frequencies within $<3$ GHz ($<0.4\%$) of design targets and *Q*-values near 100. Exact power normalizations are difficult to interpret, however, due to the difficulty of achieving precise alignments between the device ports and the VNA’s tiny WR1.0 probes. Results are consistent with a model with reduced (by ${\sim }50\times$) conductivity along the side walls due to poor lid coupling; device performance was indeed highly sensitive to the applied clamping force on the structure. These results nonetheless suggest that $R\sim 100$ resolutions are feasible and are expected to improve with cryogenic temperatures and improved wafer clamping (or bonding).

## Cryogenic Test Devices

**Fig. 5:**
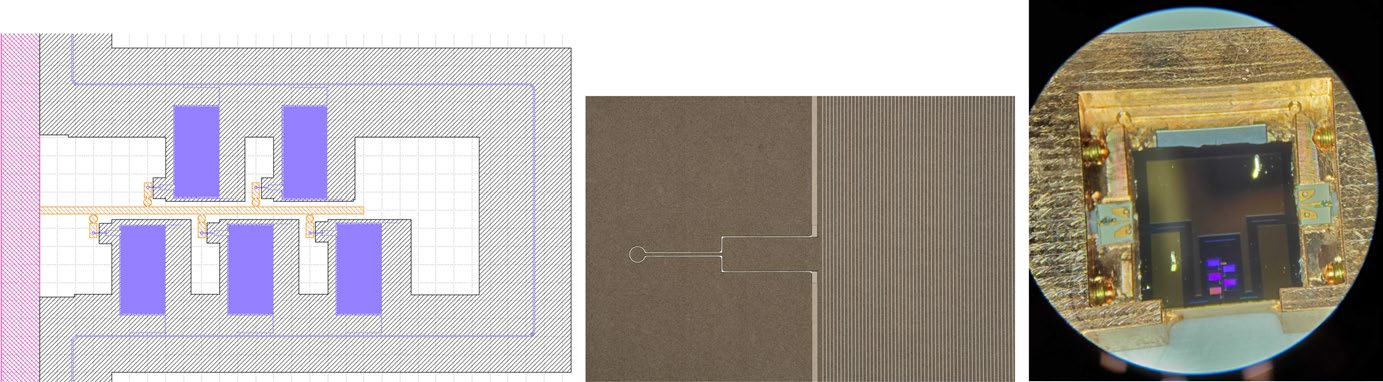
*Left:* Combined 5-channel test device masks, showing etched waveguide (*orange*), KID and readout lines (*blue*), and gold coverage on the lid (*white*). *Center:* Representative KID, showing inductor/antenna loop at left, inter-digitated capacitor at right. *Right:* Completed KID wafer mounted in housing (waveguide wafer removed); wafer is ${\sim }$14 mm across

The design of the first 5-resonator KID-coupled prototype is shown in Fig. 5 (left). The KIDs and readout lines are patterned onto the lid wafer, with coupling loops extending over each resonator; simulations indicate that high absorption efficiencies (60–95%) can be achieved throughout a 35% bandwidth [16]. The lid wafer is sputtered with gold over the waveguides, but left bare elsewhere (*gray*) to limit dissipative coupling between the KIDs and normal metal films. Wafer alignment at the necessary few-$\mu$m level has been demonstrated using a pin-and-hole scheme and an infrared microscope. This prototype KID assembly was tested with a black body optical load in a dilution refrigerator test stand at the University of Chicago. All 5 KID resonances were detected and responded to blackbody temperature changes. Spectral measurements will be made with a cryogenic FTS, which is nearly complete. See [16] for further detail on the refinement and testing of these devices.

## Discussion

We have proposed a novel on-chip spectrometer design scalable to THz frequencies, which are largely inaccessible to existing designs. We have demonstrated $Q\sim 100$ structures operating at 0.9 THz at room temperature, and all core fabrication and assembly steps have been demonstrated. Process development and fabrication are in progress for second-generation test devices.

While the initial test structures shown here were optimized for ease of fabrication and testing, with deep etching on only one wafer and simple sectoral horns, a wide variety of designs are possible within this general architecture. In particular, future work will explore designs that incorporate improved feeds and divide the waveguide channel between the two wafers, avoiding the suboptimal “corner split” employed here; this should be feasible given the alignment demonstrated in this initial work.
